# Steeling or Sensitizing? A Longitudinal Examination of How Ongoing Accumulation of Negative Life Events Affects Depressive Symptoms in Older Adults

**DOI:** 10.1093/geronb/gbab114

**Published:** 2021-06-25

**Authors:** Almar A L Kok, Jos W R Twisk, Fenneke Blom, Aartjan T F Beekman, Martijn Huisman

**Affiliations:** 1 Department of Epidemiology and Data Science, Amsterdam UMC, Vrije Universiteit, Amsterdam Public Health Institute, Amsterdam, The Netherlands; 2 Department of Psychiatry, Amsterdam UMC, Vrije Universiteit, Amsterdam Public Health Institute, Amsterdam, The Netherlands

**Keywords:** Depression, Emotion, Stress, Stressor, Within-person change

## Abstract

**Objectives:**

To examine whether (a) as people age, accumulation of negative events increases (“sensitizing”) or decreases (“steeling”) the detrimental effects of subsequent events on depressive symptoms, and (b) how particular psychosocial factors are associated with the strength of these steeling or sensitizing effects.

**Method:**

We used data from 6 measurement waves from 2,069 adults aged 55–84 (*M* = 68.0) at baseline in the Longitudinal Aging Study Amsterdam, the Netherlands. We included 18 different life events across the life course. Using hybrid multilevel models, we tested whether the effects of proximate life events (<3 years) on depressive symptoms (measured by the Center for Epidemiologic Studies Depression scale) were moderated by previous cumulative events (childhood until previous measurement wave). Additionally, we tested whether education, mastery, emotional support, neuroticism, having strong faith, and loneliness were associated with the strength of steeling/sensitizing effects.

**Results:**

Cumulative and proximate life events were independently associated with more depressive symptoms. Interaction effects indicated that the more cumulative life events, the weaker the effects of recent life events, suggesting a “steeling” effect. Unexpectedly, 3-way interaction effects showed that higher mastery and lower neuroticism were associated with weaker steeling effects. These effects were predominantly attributable to within-person changes rather than to fixed between-person differences. Results from analyses with event severity scores were similar.

**Conclusions:**

As a population, older adults appear to become more resilient against new stressors as they accumulate experience in dealing with negative life events. Findings on mastery tentatively suggest that accepting limits to one’s own control over life circumstances may foster a steeling effect.

Old age is often characterized by an increasing incidence of negative and potentially stressful life events, for example the death or severe illness of significant others (Bjorck & Thurman, 2007). It has been shown that such life events may negatively influence emotional well-being of older adults (Kraaij et al., 2002). In addition, life events that have taken place long before old age, such as the divorce of one’s parents in childhood or a spell of involuntary unemployment in adulthood, are associated with unfavorable trajectories of emotional functioning in old age (Jordanova et al., 2007; Kok et al., 2017). Accordingly, it is often found that reporting more negative life events earlier in life is associated with poorer emotional functioning in late life (Kraaij et al., 2002). These general observations indicate a need for adopting a developmental perspective in life events research (Rutter & Sandberg, 1992).

However, one important issue within this perspective concerns the precise role of earlier exposure to stressors in the impact that later negative life events have on emotional functioning (Maughan & McCarthy, 1997). There are two apparently contradicting hypotheses about this role, namely the “sensitizing hypothesis” and the “steeling hypothesis.” The former states that stress exposures make people more sensitive to later stressors, leading to stronger unfavorable effects of proximate stressors on emotional functioning in people who have accumulated more earlier exposure. The latter predicts that gathering experience with stressors may enable people to better cope with later stressors, and therefore reduces the unfavorable effect of later stressors on emotional functioning. One possibility uniting both hypotheses is that individuals differ in the extent to which they become “sensitized” or “steeled” from exposure to adverse experiences, and that this variation depends on third factors such as individual coping resources and social support (Stroud, 2018; Sutin et al., 2010; Wingenfeld et al., 2009).

Empirical evidence on the extent to which these hypotheses hold true in the general older population and on the characteristics that may be associated with interindividual heterogeneity in sensitizing and steeling effects is scarce (Liu, 2015; Rutter & Sandberg, 1992). Therefore, in this 16-year longitudinal study, we examine how ongoing exposure to negative life events affects depressive symptoms in older adults, and whether resources such as emotional support and perceived control over life are associated with sensitizing and steeling effects.

## Theory and Evidence on Sensitizing versus Steeling Effects

According to the stress sensitization model, exposure to stress can increase the sensitivity to future stressors; older adults that have in the past been exposed to severe stressors are expected to experience more detrimental effects of proximate stressors in old age on emotional functioning, particularly on the risk of major depression, than those not exposed (Monroe & Simons, 1991; Stroud, 2018). The model posits that earlier stressors may leave lasting vulnerabilities that can negatively affect emotional functioning, even if proximate stressors are of relatively low severity (Stroud, 2018). Few studies have examined the mechanisms that are responsible for this sensitizing effect. Suggested mechanisms are neurobiological (e.g., hypothalamic–pituitary–adrenal axis dysregulation or cortisol reactivity), or cognitive/psychological (e.g., alterations in personality or in cognitive schema that involve depressogenic patterns of thinking [Stroud, 2018]). Nevertheless, there is substantial evidence that supports the general premise of the sensitizing hypothesis (e.g., Hammen et al., 2009; McLaughlin et al., 2010; Tennant, 2002).

In contrast to the sensitizing hypothesis, the “steeling hypothesis” predicts that individuals who gather experience in coping with stressful life events may develop strategies or psychological buffers that protect against the negative effects of potential future life events (Rutter, 1985). In the context of life events, the “steeling hypothesis” would predict that as exposure to negative life events accumulates, the effects of proximate events on emotional functioning decline. However, it is argued that stressors should be of moderate severity, as severe stressors may be too overwhelming to result in a positive learning effect, and stressors of low severity do not pose a sufficient challenge (Liu, 2015).

We identified only two studies that have examined steeling effects in relation to negative life events. Seery and colleagues (2010) showed in an internet panel study (*n* = 2,398; maximum follow-up = 3 years; mean age = 49) that the negative effects of proximate life events on mental health (including distress, life satisfaction, and posttraumatic stress symptoms) were lowest in those with moderate exposure to earlier life events, as opposed to no or high exposure (Seery et al., 2010). Shapero and colleagues (2015) demonstrated similar steeling effects in adolescents (*n* = 163; maximum follow-up = 3 years; mean age = 13), using depressive symptomatology as outcome (Shapero et al., 2015). These studies thus suggest that exposure to stressors may to some extent “inoculate” individuals against the impact of later stressors, but only if the early exposure is of moderate severity.

## The Potential Role of Psychosocial Resources

Although at first glance the sensitizing and steeling hypotheses seem contradictory, they may in fact be compatible. Whether sensitizing or steeling effects occur may in part depend on the psychological and social resources that individuals bring along to cope with negative events (Rutter & Sandberg, 1992). Thus, accumulation of stressful events may result in steeling effects in some individuals while it is sensitizing in others, and these individual differences might be partly explained by differences in the severity of exposures and differences in psychosocial resources.

Models of stress sensitization posit that endogenous (e.g., personality characteristics) and exogenous (e.g., social support) factors may influence the extent to which individuals build resilience or vulnerability towards negative events across the life course (Rodgers, 1991). Studies have shown that higher perceived control over one’s life, more social support, stronger religiousness, and higher educational attainment are associated with reduced negative effects of stressful life events on emotional functioning (Bjorck & Thurman, 2007; Jopp & Schmitt, 2010; Lim et al., 2015). Additionally, personality factors such as (low) neuroticism may have similar protective effects, although not all previous studies found such effects (Brown & Rosellini, 2011; Spinhoven et al., 2011). It is unknown whether such factors can also predict the extent to which individuals can positively learn from ongoing exposure to negative life events. That is, whether these factors can distinguish persons in whom accumulation of negative life events tends to decrease the effect of subsequent events (i.e., “steeling”) from those in whom accumulation of events increases the effect (i.e., “sensitizing”).

## The Current Study

This study aims to answer two questions. First, whether on average, there is a “steeling” or “sensitizing” effect of previous cumulative negative life events on the impact of proximate events on depressive symptoms. The steeling hypothesis (H1a) states that the impact of proximate events decreases as exposure to previous events increases, while the sensitizing hypothesis (H1b) states that the impact of proximate events increases. Second, whether the strength of sensitizing or steeling effects depends on individual differences in psychosocial resources. We hypothesize that older adults with more psychosocial resources (e.g., higher mastery and more emotional support) experience stronger steeling effects of cumulative events on proximate events (H2). The mechanisms implied by these questions are approached as developmental processes, and are therefore studied longitudinally using 16-year prospective data from a population-based study of Dutch adults aged 55 and older. Accordingly, in analyzing these data, we distinguish between-person differences from within-person changes.

## Method

### Study Sample

We used data from the Longitudinal Aging Study Amsterdam (LASA; Huisman et al., 2011), which is based on a random sample of adults aged 55–84 years at baseline from 11 municipalities in the Netherlands, selected to provide national representativeness in terms of demography and cultural background. At baseline in 1992/1993, LASA included 3,107 participants. We used data from the baseline measurement and five follow-up measurements in 1995/1996, 1998/1999, 2001/2002, 2005/2006, and 2008/2009. Data on the occurrence of life events before baseline were retrospectively reported in the baseline measurement. However, because we also required at least one report of subsequent proximate life events—which was asked from the second measurement wave onwards—we included only participants who participated in the second wave in 1995/1996 (*n* = 2,545). Furthermore, we included only participants who provided data on all variables (life events, depressive symptoms, and all covariates) on at least one measurement occasion since 1995/1996 (*n* = 2,069; 8,161 observations; mean age = 68.0, *SD =* 8.4; 53% female). Further sample attrition and missing data on the third to sixth measurement waves were handled by maximum likelihood estimation.

### Measures

#### Depressive symptoms

Depressive symptoms were measured with the Center for Epidemiologic Studies Depression scale (CES-D; Radloff, 1977). The CES-D contains 20 items on experienced depressive symptomatology during the past week, which cover key components associated with clinical depression: depressed mood, feelings of helplessness, hopelessness, worthlessness and guilt, retarded movement, sleep disturbance, and loss of appetite (Radloff, 1977). Items are answered on a 4-point scale (scores 0–3) and add up to a scale ranging from 0 to 60. Examples are [during the past week…] “I talked less than usual,” and “I felt my life is a failure.” In LASA, the CES-D has shown high reliability and good criterion validity (Beekman et al., 1997). Cronbach’s alpha in our final sample ranged between .86 and .87 across waves. Missing items were imputed with the mean of available items. We had to log-transform depressive symptoms because of violation of the normally distributed residuals and linearity assumptions of linear regression. Depressive symptoms across Waves 2–6 were used as dependent variables.

#### Proximate and cumulative life events

Proximate events consisted of a checklist of 12 life events that was asked from the second measurement wave onwards. For each event, participants indicated whether they experienced this event since the previous measurement wave, that is, within a 3- to 4-year period. The list included the death of the participant's father, mother, brother, sister, son, daughter, or grandchild; severe illness of the partner or a relative; becoming a victim of crime; having a severe conflict; or having financial problems. In addition to this list, we used longitudinal data on partner status to include whether the participant became divorced or widowed since the previous wave, resulting in a total of 14 possible life events. Because of violation of the linearity assumption, we categorized proximate events into 0, 1, 2, and 3 or more.

The checklist of 12 proximate life events was not asked at baseline. Therefore, for assessing cumulative exposure to life events before the baseline measurement we used a selection of eight events that were asked in different sections in the baseline interview, as described in a previous study (Kok et al., 2017). We included whether the participant ever experienced divorce of parents or severe discord between parents before age 18; death of the father and death of the mother (at any age); divorce; widowhood; death of a child; ever becoming involuntarily unemployed for at least 4 months; and ever becoming occupationally disabled for at least 3 months.

To express the total cumulative exposure to life events up to each follow-up measurement, from the second wave onwards, we added the number of proximate events to the number of cumulative events occurring before the *previous* wave, ensuring that no life events were counted twice. Therefore, on average, the number of cumulative events increased with each new measurement wave.

#### Psychosocial resources

Our selection was based on the broad categories of psychosocial resources shown to potentially moderate the effect of life events on depressive symptomatology. These domains were socioeconomic resources, social support, and psychological characteristics (Bjorck & Thurman, 2007; Brown & Rosellini, 2011; Jopp & Schmitt, 2010; Lim et al., 2015; Spinhoven et al., 2011). From each domain, we included one or more variables that we had available for multiple measurement waves.

For measuring the domain of social support, we included the frequency of receiving emotional support, and loneliness. The amount of emotional support received was based on an extensive social network delineation questionnaire (van Tilburg, 1998). For the nine social network members that were indicated by the participants as their most important contacts, participants reported how often they talked with them about personal experiences and feelings. Response categories were never (1), seldom (2), sometimes (3), and often (4). Participants who indicated to have less than nine network members could not reach the maximum score; those who indicated to have no network members received a score of 0. The answers were summed, resulting in a range from 0 to 36 (van Tilburg, 1998).

Loneliness was based on the 11-item de Jong-Gierveld Loneliness scale (de Jong-Gierveld & Kamphuis, 1985). We included this measure as a qualitative measure of perceived social support to complement the more quantitative measure of the frequency of receiving emotional support. Example items are “I miss having a really close friend” and “I find my circle of friends and acquaintances too limited.” Answer categories are yes (0), more or less (1) and no (2). After reverse coding positively worded items, and dichotomizing the items, where answer categories (1) and (2) indicated loneliness, items were summed to a score of 0–11 (de Jong-Gierveld & Kamphuis, 1985). Cronbach’s alpha in our final sample ranged between 0.80 and 0.82 across waves.

For the domain of psychological characteristics, we included mastery as a measure of perceived control over life, which is relevant in the context of the often uncontrollable nature of the events in included in our study (Jopp & Schmitt, 2010), and neuroticism as an indicator of emotional stability (Brown & Rosellini, 2011). Mastery was based on a five-item version of the Pearlin Mastery Scale, with response categories from totally disagree (1) to totally agree (5), and a total scale score of 5–25 (Pearlin et al., 1981). Example items are “I have little control over the things that happen to me,” and “There are few things I can do to change important things in my life.” Cronbach’s alpha in our final sample ranged between .72 and .75 across waves. Missing items were imputed with the mean of available items. Neuroticism refers to a general tendency to experience emotional distress and react with distress to events and situations (Costa & McCrae, 1992), and was measured by a shortened version (15 instead of 20 items) of the Dutch Personality Questionnaire (Barelds & Luteijn, 2002). The 15 items asked to what extent specific statements applied to the participants; answer categories were 0 (does not apply to me), 1 (don’t know), and 2 (applies to me). Examples are “I often have bad moods without knowing why” and “I often worry about little things.” Answers are summed to a scale score of 0–30. Cronbach’s alpha in our final sample ranged between .81 and .82 across waves. Missing items were imputed with the mean of available items. Because neuroticism was measured only at the first four waves and had relatively many missing responses because it was asked in a postal questionnaire rather than the main face-to-face interview, we computed the mean score across all available waves, and regarded neuroticism as a fixed factor.

For the domain of socioeconomic resources, we included education, which was originally asked in nine categories that we recoded to the nominal years it takes to complete that level of education, ranging from 5 to 18 years.

Finally, religiosity is seen as a coping resource in the context of negative life events. The importance of religiosity in the lives of the participants was approximated by an item indicating whether the participant felt that “strong faith” was one of the three most important aspects in his or her life (1 = yes, 0 = no).

### Analytic Procedure

We generated descriptive statistics of all study variables at each measurement wave, and examined differences in baseline variables between those included in the analyses and those excluded. Then, in a stepwise procedure, we estimated increasingly complex linear mixed models based on six repeated observations nested within individuals (for a graphical representation of the model guiding the analyses, see Supplementary Figure S1).

We examined conventional mixed models and hybrid mixed models. The conventional models provide composite effects that express a mixture of within- and between-person effects. The hybrid models disaggregate the composite effect into within-person and between-person effects (Curran & Bauer, 2011; Hoffman & Stawski, 2009). With this method, we are able to demonstrate effects of (the accumulation of) life events on within-person changes in depressive symptoms over time, as well as effects on the mean level of depressive symptoms across the observation period. This disaggregation is only possible with factors that are able to change over time. All continuous variables and depressive symptoms were standardized to *z*-values based on all available observations, except for cumulative life events.

First, we estimated a model including only the proximate and cumulative events variables to examine their independent associations with depressive symptoms. For descriptive purposes, we also estimated the associations between each psychosocial resource and depressive symptoms in separate models. Second, to analyze whether, on average, previous exposure to life events would be associated with stronger or weaker effects of proximate events on depressive symptoms (H1a and H1b), we added a two-way interaction effect between proximate and total accumulated life events. A negative interaction effect would indicate, on average, a “steeling effect,” while a positive interaction would indicate a sensitizing effect. Third, to analyze whether psychological factors are associated with the strength of sensitizing or steeling effects (H2), we expanded the models with a three-way interaction effect between education, strong faith, emotional support, mastery, loneliness, and neuroticism and the interaction of proximate and total accumulated events. These effects were estimated in separate models including a single psychosocial resource. We also examined three-way interaction effects between age and sex and the Cumulative * Proximate events interaction. All models were adjusted for age and sex and estimated using maximum likelihood procedures to handle missing data. In addition, we adjusted for depressive symptoms at the LASA baseline measurement in 1992/1993, to examine whether life events were associated with the participant-mean level of depressive symptoms across follow-up waves, independent of the initial level of symptoms. Within-person effects are not affected by this adjustment.

As a sensitivity analysis to address the fact that the included life events may differ in severity, we estimated all models again using a severity sum score instead of a simple count of life events, based on rankings developed in research by Hobson and colleagues (1998). These rankings were derived in a sample of *n =* 3,122, representative of the U.S. population, stratified for age. We used rankings from the group aged 65 and over. For details on the scoring, see Supplementary Material. Furthermore, to assess potential bias due to selective attrition, we repeated main analyses excluding participants who deceased during follow-up (included *n* = 1,045) and excluding participants with incomplete data at any wave (included *n* = 573).

## Results

### Descriptive Statistics

At baseline, the participants were on average 68 years old (*SD* = 8.4), and 53% was female. The average score on the CES-D at baseline in 1992/1993 was 7.2 (*SD* = 7.2; not log-transformed), and participants reported on average 2.5 (*SD* = 0.9) life events occurring before baseline (Table 1). The cumulative number of life events gradually increased to on average 6.8 (*SD =* 2.5) at the sixth measurement wave. The number of proximate life events was about equal between all waves (not shown in Table 1). For events before baseline (total *n* = 1,780), bereavement (25.3%) and death of a parent (14.5%) were most frequently mentioned, yet the latter was not significantly associated with depressive symptoms (see Supplementary Figure S2). For proximate events (total *n* = 7,494), severe illness of other than spouse was most frequently reported (43.9%) and divorce least frequently (<0.1%). In addition to this event, bereavement, conflict, victim of crime, death of a son or daughter, and financial problems were significantly associated with depressive symptoms.

**Table 1.** Descriptive Statistics of Included (*n* = 2,069) vs Excluded (*n* = 476) Participants

	Included (*n* = 2,069)		Excluded (*n* = 476)		
Variable	*n*	Mean (*SD*) or %	*n*	Mean (*SD*) or %	*p* value
Baseline age (55–84 years)	2,069	68.0 (8.38)	476	73.8 (8.06)	<.001
Sex (female)	2,069	52.6	476	56.5	.13
Depressive symptoms (0–60)	2,068	7.2 (7.17)	476	9.6 (8.75)	<.001
Proximate events between baseline and first follow-up^a^	2,004		198		.22
0	555	27.7	60	30.3	
1	778	38.8	73	36.9	
2	480	24.0	39	19.7	
3+	191	9.5	26	13.1	
Cumulative events before baseline (1992)	2,069	2.5 (0.92)	397	2.7 (0.94)	.002
Up to 1995	2,004	3.7 (1.34)	n/a		
Up to 1998	1,623	4.7 (1.77)	n/a		
Up to 2002	1,227	5.8 (2.26)	n/a		
Up to 2005	845	6.8 (2.50)	n/a		
Education	2,069	9.1 (3.32)	473	8.0 (3.13)	<.001
Mastery^a^	2,008	17.5 (3.27)	207	15.8 (3.59)	<.001
Emotional support^a^	2,001	21.2 (8.06)	196	17.5 (9.47)	<.001
Strong faith (% yes)^a^	1,926	18.6	93	25.8	.08
Loneliness^a^	2,024	2.1 (2.55)	254	3.5 (2.94)	<.001
Neuroticism	2,069	5.9 (5.31)	310	7.5 (6.77)	<.001

*Notes*: n/a = not applicable.

^a^At first follow-up in 1995/1996; other variables were measured at baseline in 1992/1993.

### Sample Selectivity

The included sample (*n* = 2,069) was younger, had fewer cumulative life events before baseline, higher education and mastery, and lower loneliness and neuroticism than the excluded sample (*n* = 476; Table 1). There was no statistically significant difference in sex composition, the number of proximate events, or the percentage indicating strong faith as an important aspect of life between the included and excluded groups.

### Direct Associations With Depressive Symptoms

Adjusted for age, sex, and baseline depressive symptoms, proximate events showed a dose–response relationship with depressive symptoms; the composite effect for one versus no proximate events on the log-transformed and standardized depressive symptoms scale was *b =* 0.08 (95% confidence interval [CI] = 0.04–0.13); for two versus no events *b* = 0.18 (CI = 0.13–0.24); and for three or more events *b* = 0.32 (CI = 0.24–0.39; Table 2). Except for the between-person effect of one proximate event, all effects on depressive symptoms were statistically significant, indicating that effects on *changes in* depressive symptoms as well as effects on the mean level of depressive symptoms across all waves contributed to the composite effect. Independent from effects of proximate events, cumulative events also showed a positive composite effect on depressive symptoms (*b =* 0.07, CI = 0.06–0.08), representing significant effects at the within-person and between-person level.

**Table 2.** Associations Between Life Events and Psychosocial Resources, and Depressive Symptoms (Log-Transformed and Standardized), Adjusted for Age, Sex, and Baseline Depressive Symptoms

		Composite effect			Within−person effect			Between−person effect		
Variable	Obs	*B*	95% CI	*p*	*B*	95% CI	*p*	*B*	95% CI	*p*
Proximate events (ref. = 0)	6,345									
1		0.08	0.04–0.13	<.001	0.10	0.05–0.15	<.001	0.05	−0.06 to 0.17	.36
2		0.18	0.13–0.24	<.001	0.22	0.16–0.28	<.001	0.15	0.02–0.28	.02
3+		0.32	0.24–0.39	<.001	0.38	0.29–0.47	<.001	0.21	0.04–0.38	.01
Cumulative events	6,345	0.07	0.06–0.08	<.001	0.08	0.07–0.09	<.001	0.04	0.01–0.06	.001
Education^a^	7,023	−0.03	−0.06 to 0.003	.07	n/a (fixed factor)					
Mastery^a^	6,685	−0.32	−0.34 to −0.30	<.001	−0.27	−0.29 to −0.24	<.001	−0.38	−0.41 to −0.34	<.001
Emotional support^a^	6,642	0.01	−0.01 to 0.03	.31	0.03	−0.002 to 0.06	.07	−0.02	−0.06 to 0.02	.31
Strong faith	6,485	0.03	−0.03 to 0.09	.31	0.02	−0.06 to 0.11	.58	0.03	−0.06 to 0.12	.49
Loneliness^a^	6,980	0.28	0.25–0.30	<.001	0.25	0.22–0.28	<.001	0.30	0.27–0.34	<.001
Neuroticism^a^	7,023	0.30	0.27–0.33	<.001	n/a (fixed factor)					

*Notes*: n/a = not applicable; obs = observed.

^a^Standardized.

Lower mastery, higher loneliness, and higher neuroticism were significantly associated with more depressive symptoms, both within-person and between-person. We found no significant associations between education, emotional support and indicating strong faith as an important aspects of life, and depressive symptoms.

### Two-Way Interaction Effect Between Cumulative and Proximate Events

At the composite level, the interaction effects between cumulative events and proximate events were negative, suggesting a steeling effect of cumulative events (Table 3 and Supplementary Figure S3). However, only the effect at two proximate events reached statistical significance. Nevertheless, the hybrid multilevel results showed that at the within-person level, there was a clear negative (steeling) effect at all levels of proximate events, whereas the between-person interaction effect was positive, and significant for one and three proximate events. These opposite effects indicate that over time, an increase in the total accumulated number of events was associated with weaker effects of additional proximate events on depressive symptoms. However, regardless of these within-person changes over time, persons with more cumulative events who experience one or three or more proximate events tend to have a higher mean level of depressive symptoms across the observation period than persons with less cumulative events.

**Table 3.** Interaction Effect Between Proximate and Cumulative Life Events on Depressive Symptoms (Log-Transformed and Standardized), Calculated With Linear Mixed Models, Adjusted for Age, Sex, and Baseline Depressive Symptoms

	Composite effect			Within-person effect			Between-person effect		
Variable	*B*	95% CI	*p*	*B*	95% CI	*p*	*B*	95% CI	*p*
Cumulative and proximate events									
Proximate events (ref. = 0)									
1	0.12	0.02–0.22	.02	0.22	0.11–0.33	<.001	−0.19	−0.45 to 0.06	.14
2	0.32	0.21–0.44	<.001	0.46	0.32–0.59	<.001	0.06	−0.23 to 0.35	.70
3+	0.41	0.25–0.56	<.001	0.64	0.46–0.82	<.001	−0.26	−0.64 to 0.11	.16
Cumulative events	0.08	0.07–0.10	<.001	0.12	0.09–0.14	<.001	−0.01	−0.07 to 0.04	.59
Proximate * Cumulative									
1	−0.01	−0.03 to 0.01	.40	−0.03	−0.05 to −0.003	0.03	0.08	0.01–0.15	.03
2	−0.03	−0.06 to −0.01	.01	−0.05	−0.08 to −0.03	<.001	0.03	−0.05 to 0.11	.54
3+	−0.02	−0.05 to 0.01	.17	−0.06	−0.09 to −0.02	0.001	0.14	0.04–0.23	.004

### Three-Way Interaction Effects Between Psychosocial Resources, Age, Sex, and Life Events

**Figure 1.**
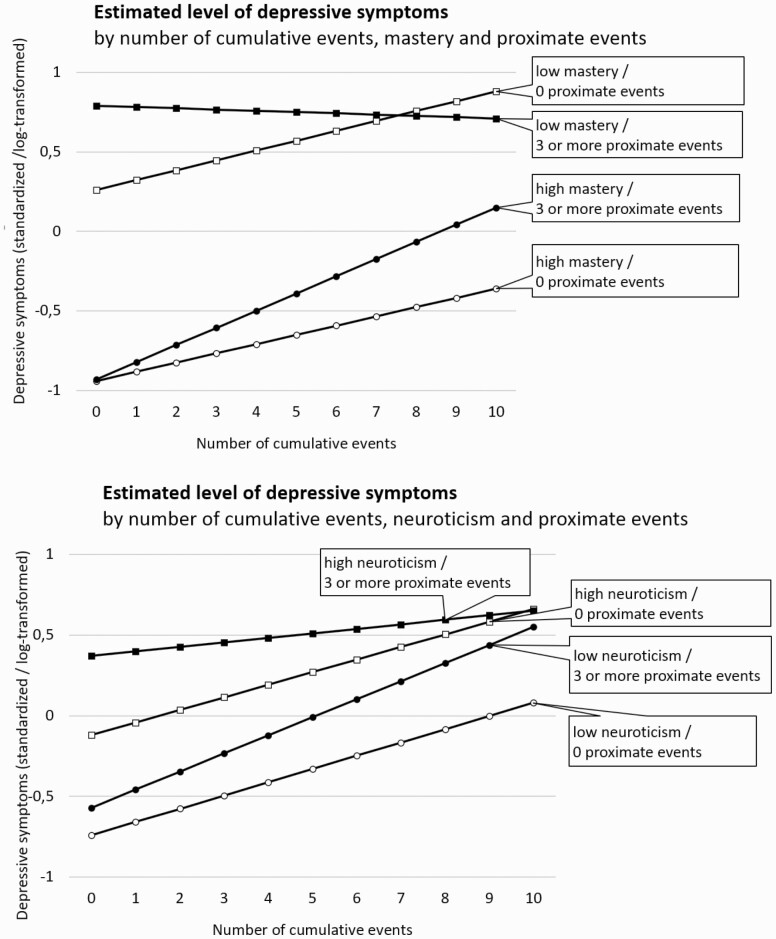
Graphical representation of the significant composite three-way interaction effects between mastery/neuroticism, cumulative life events, and three or more proximate life events. The *y*-axis represents the level of depressive symptoms (weighted average across all measurement waves, log-transformed and standardized). The *x*-axis represents the number of cumulative life events. Graphs indicate that for persons with low mastery (−2 *SD*) and high neuroticism (+1 *SD*), the difference in depressive symptoms between those with no vs three or more proximate events decreases as cumulative events increase, suggesting a “steeling effect” of cumulative events. In contrast, for those with high mastery (+2 *SD*) and low neuroticism (−1 *SD*), the difference in depressive symptoms between those with no vs three or more proximate events increases as cumulative events increase, suggesting a “sensitizing effect” of cumulative events. *Note:* Models also included all main effects and two-way interaction effects.

For mastery and neuroticism, we found three-way composite interaction effects with a *p* value below .05, specifically for three or more proximate events (Figure 1 and Table 4; estimates are based on models also including all main effects and two-way interaction effects). The effects suggested that the steeling effect of cumulative events was weaker with higher mastery and lower neuroticism. Figure 1 illustrates this; it shows that at each particular moment in time, for persons with low mastery (−2 *SD*) and high neuroticism (+1 *SD*), the difference in the level of depressive symptoms between those without and those with three or more proximate events decreases as cumulative events increase, suggesting a “steeling effect” of cumulative events for those with lower mastery and higher neuroticism. In contrast, for those with high mastery (+2 *SD*) and low neuroticism (−1 *SD*), the difference in depressive symptoms between those without versus those with three or more proximate events *increases* as cumulative events increase, suggesting a “sensitizing effect” of cumulative events for those with higher mastery and lower neuroticism. The hybrid models suggested that these effects were mainly due to within-person changes, as only the within-person effect was statistically significant. Supplementary Tables S6 and S7 show all coefficients included in these models.

**Table 4.** Models With Statistically Significant Three-Way Interaction Effects on Depressive Symptoms (Log-Transformed and Standardized), Calculated With Linear Mixed Models, Adjusted for Age, Sex, and Baseline Depressive Symptoms^a^

	Composite effect			Within-person effect			Between-person effect		
Variable	*B*	95% CI	*p*	*B*	95% CI	*p*	*B*	95% CI	*p*
Mastery									
Proximate events (ref. = 0)									
1	0.07	−0.02 to 0.17	.14	0.17	0.06–0.28	.002	−0.14	−0.37 to 0.10	.26
2	0.25	0.13–0.36	<.001	0.38	0.25–0.51	<.001	0.10	−0.16 to 0.37	.44
3+	0.27	0.12–0.42	<.001	0.49	0.31–0.67	<.001	−0.13	−0.48 to 0.21	.45
Cumulative events	0.06	0.04–0.08	<.001	0.09	0.07–0.11	<.001	0.01	−0.04 to 0.05	.80
Mastery	−0.30	−0.38 to −0.22	<.001	−0.19	−0.29 to −0.10	<.001	−0.33	−0.50 to −0.16	<.001
Mastery * Proximate * Cumulative events									
1	−0.004	−0.03 to 0.02	.70	−0.003	−0.03 to 0.02	.81	0.03	−0.04 to 0.10	.40
2	0.02	−0.004 to 0.05	.10	0.03	−0.002 to 0.05	.07	0.03	−0.04 to 0.10	.38
3+	0.03	<0.001–0.06	.05	0.04	0.01–0.07	.02	0.05	−0.03 to 0.13	.24
Neuroticism									
Proximate events (ref. = 0)									
1	0.11	0.01–0.21	.03	n/a (fixed factor)					
2	0.30	0.18–0.41	<.001						
3+	0.33	0.18–0.49	<.001						
Cumulative events	0.08	0.06–0.10	<.001						
Neuroticism	0.31	0.23–0.40	<.001						
Neuroticism * Proximate * Cumulative events									
1	−0.02	−0.04 to 0.01	.15						
2	−0.01	−0.03 to 0.02	.56						
3+	−0.04	−0.07 to −0.01	.01						

*Notes*: n/a = not applicable. Details are reported in Supplementary Tables S6 and S7.

^a^All two-way interaction effects were included in the models.

### Sensitivity Analyses

We found no substantial differences between the results from models using life events severity scores and the results from models using counts of life events (Supplementary Tables S1–S5). The sensitivity analyses in participants who did not decease or dropped out for other reasons during follow-up showed somewhat stronger main effects of proximate events on depressive symptoms, but the point estimates of the interaction effects were similar, indicating that our main findings concerning steeling and sensitizing effects were adequately robust to attrition (Supplementary Table S8). Nevertheless, in these smaller samples, the three-way interaction effects were no longer statistically significant, suggesting that they are relatively weak.

## Discussion

This study examined whether ongoing accumulation of exposure to negative life events has a “steeling” or “sensitizing” effect on depressive symptoms in the general Dutch older population. Specifically, we tested whether the effect of proximate life events on depressive symptoms became stronger or weaker as older adults were exposed to an increasing number of negative life events, and whether psychosocial resources were associated with the strengths of these steeling or sensitizing effects. The majority of the life events involved the death or severe illness of one’s spouse or close others.

Our central finding is twofold and stems from our disaggregation of within-person and between-person effects. We found that as people age, ongoing accumulation of negative life events tends to decrease the impact of new events on depressive symptoms, suggesting a steeling effect, supporting hypothesis 1a. However, analysis of between-person differences showed that the total history of exposure to negative life events matters: at any given time point, despite the steeling effect, older adults with more cumulative events who experience new negative events tend to have a higher overall level of depressive symptoms across old age. Furthermore, we examined whether individual psychosocial resources influenced the extent to which older adults experience steeling or sensitizing effects. We found that higher mastery and lower neuroticism were associated with a weaker steeling effect, at least when confronted with a high number of proximate events, contradicting hypothesis 2. The findings were comparable when we used event severity scores instead of event counts.

### Steeling and Severity of Exposure

Our main finding is broadly in line with one similar study (Seery et al., 2010), which found a smaller effect of proximate life events in persons with moderate exposure to earlier life events. However, Seery and colleagues (2010) also found that this steeling effect was diminished at high levels of earlier exposure, suggesting a nonlinear interaction effect between earlier and proximate exposure. We found no evidence for this nonlinear effect. Furthermore, we replicated our analyses with severity scores, but this did not change our findings based on event counts.

Our study was the first to statistically disentangle within- from between-person effects of cumulative and proximate events. The notion that the steeling hypothesis is predominantly about within-person changes was largely supported by the results from these analyses. The direction of the composite within/between effects was consistent with the within-person effects, and the latter clearly demonstrated that *increases in* exposure to life events tended to dampen the effect of subsequent events over time. Nevertheless, despite this steeling effect, proximate events still had an independent positive effect on depressive symptoms, indicating that steeling cannot be equated to “stress inoculation” (Dienstbier, 1989); it seems unlikely that persons could become immune to stressors.

### Mechanisms Behind Steeling and Sensitizing Effects

There seems to be much speculation but little empirical evidence on the mechanisms behind steeling effects (Liu, 2015). In our study, we assessed whether factors reflecting emotion regulation, coping, and the quantity (support) and quality (loneliness) of interpersonal relationships influenced the strength of the steeling effect. Against expectations, we found no influence of emotional support, loneliness and religiosity, and the effects of mastery and neuroticism indicated that the steeling effect was weaker in older adults with a stronger internal locus of control and higher emotional stability.

For mastery, one explanation may relate to the relative uncontrollability of the life events included in our study, for example, bereavement or illness of significant others. For individuals who endorse a strong sense of personal control over their lives, being confronted with uncontrollable losses may present a discrepancy between reality and internal values of control. For persons who perceive themselves to be in control of their lives, this discrepancy might elicit more negative affect than in negative situations in which they experience more control. In situations that are outside the individual’s control, successful self-regulation may require acceptance of constraints on one’s life and social environment rather than active attempts to intervene on one’s life circumstances (Kok et al., 2018; Wrosch & Freund, 2001). For persons with a strong internal locus of control, it may be harder to accept negative life events such as those included in our study. Furthermore, qualitative research suggests that reinterpreting the meaning of previous and recent events in the context of one another is essential to developing resilience, and that people readjust their expectations in light of changing circumstances (Hildon et al., 2008). In the context of increasing exposure to negative life events in old age, this readjustment might involve a *lowering* of one’s sense of mastery. This would be in line with the *within-person* effects of mastery that indicated stronger steeling effects with lower mastery over time, and it is in line with the idea that mastery is dynamic in late life (Pearlin et al., 2007). Nevertheless, these results should be interpreted in the context of the finding that the general level of depressive symptoms in persons with lower exposure to life events and higher levels of mastery was substantially lower throughout the observation period.

The finding that lower neuroticism was associated with a weaker steeling effect is hard to explain from the previous literature. It may be important to consider that neuroticism (and to a lesser extent mastery) is typically highly correlated with depressive symptoms. It could be that in persons with low neuroticism, the scope for negative life events to severely increase the level of depressive symptoms is generally also low to begin with, because they tend to respond with less distress to such events. This would imply that the scope for reducing the effects of additional events on depressive symptoms is also smaller than in those with high neuroticism. Given the lack of empirical studies on this possibility in the context of ageing and life event accumulation, it is clear that future studies are needed to replicate our present findings, and examine in more detail the possibility that while a higher sense of control and emotional stability are generally associated with better mental health, their relationship over time may be different when facing multiple stressors.

### Implications

Most older adults appear to be able to use their accumulating life experiences—including negative ones—to reduce the impact of new stressors on their well-being. This provides nuance to a view of old age as a period of inherent increasing vulnerability. Still, we also showed that despite the steeling effect that may occur over time, exposure to life events is associated with a higher overall level of depressive symptomatology across old age—and thus a point of concern. Furthermore, while indicators of effective self-regulation such as mastery and neuroticism are generally associated with better well-being and a lower impact of proximate life events, our findings indicate that in the presence of accumulating exposure to negative events, such resources may become less effective. The clinical implication is that there may be limits to the ability of older adults to draw from these mostly beneficial individual coping strategies. However, this implication should be interpreted with caution, as it is hard to evaluate the size and practical meaning of the complex interaction effects that we found in this population-based sample. The majority of the effects we found are likely to play out within the normal range of depressive symptomatology and may not be directly translatable to clinical practice.

### Strengths and Limitations

To our knowledge, this study was the first to examine the steeling effect in the context of ongoing exposure to negative life events in a population-based sample of older adults. Strengths of the present study include the large sample size, the long follow-up, the inclusion of several psychosocial resources as effect modifiers, and the use of contemporary statistical methods to examine longitudinal effects, including an examination of the relative contributions of within- and between-person effects. Furthermore, the ability to use maximum likelihood estimation may have reduced bias due to missing follow-up data and selective attrition. As such, the study not only provides new nuanced empirical evidence on the steeling effect but also generates entry points for future studies, particularly concerning the role of perceived control in coping with negative life events in later life.

Notable limitations to the study are, first, the fact that we had no individual information on the perceived severity of the life events, which reduced our ability to test the hypothesized dependence of the steeling effect on the severity of the stressor. Nevertheless, we observed that severe illness and death of significant others represented the majority of the events and that various types of frequent and less frequent events (e.g., parental problems in childhood and financial problems) were associated with depressive symptomatology, and are therefore likely to contribute to the observed steeling effects. Also, our results were similar when using severity scores based on normed data from a large population-based sample (Hobson et al., 1998). Furthermore, to keep the amount and complexity of the results manageable and focus on overall accumulation of exposure to life events, we did not distinguish between types and timing of events. We consider these aspects to be important for future studies. Second, due to the selection criteria for the present analysis, our results may be less generalizable to very old and lower-educated persons, who have higher drop-out rates and were less likely to participate in at least the first two LASA measurement waves, although possible bias due to missing follow-up data in the included sample was minimized by using maximum likelihood estimation. Third, for statistical reasons, we had to log-transform depressive symptoms. While back-transforming specific predicted values is possible, regression coefficients can only be interpreted in the log-transformed model. This means that while the general conclusions about the direction of effects are valid, any conclusions about their size and potential clinical relevance are tentative. Replicating the current analyses in a sample with more normally distributed depressive symptoms scores—for example, a clinical sample—could be relevant.

## Conclusion

Over time, accumulating experience in dealing with negative life events may partly ameliorate the deleterious effects of new negative events on emotional functioning in old age, yet those with high exposure to negative life events still face higher overall levels of depressive symptomatology. Differences in perceived control and emotional stability may partly explain individual differences in the steeling effect, and our results suggest that the effect is stronger for those with lower levels of mastery and higher levels of neuroticism. The buffering effects of these psychological resources may thus be less effective in the face of high exposure to negative life events. The results indicate a need for future studies to help improve our understanding of how the capacity for learning from negative experiences may be harnessed to retain high levels of well-being and prevent depression in old age.

## Supplementary Material

gbab114_suppl_Supplementary_Materials
