# Supplementary material for: Steeling or Sensitizing? A Longitudinal Examination of How Ongoing Accumulation of Negative Life Events Affects Depressive Symptoms in Older Adults
Source: J Gerontol B Psychol Sci Soc Sci. 2021 Jun 25;76(10):2041–53. doi: 10.1093/geronb/gbab114 (PMC8599083; doi:10.1093/geronb/gbab114)

Supplement to: Steeling or sensitizing? A longitudinal examination of how ongoing accumulation of negative life events affects depressive symptoms in older adults

Core analyses with severity scores instead of count of events

Severity scores obtained from Hobson et al. (1998). Stressful life events: a revision and update of the social readjustment rating scale. *International Journal of Stress Management*, *5*(1).

Scores are based on Table 3 in the Hobson et al. (1998) paper, which categorizes in age groups. We used the scores from the >=65 years group, because the baseline age in our sample is about 68 years and they are followed up to about 80 years.

Conversion table:

Table S1. Conversion table of life events from count (yes/no) into severity scores based on Hobson et al. (1998)

Life event	Hobson et al. category	Score	Remarks
**Events before baseline**			
Parental problems in childhood-	42.Domestic violence/sexual abuse	65.9	Closest category description
Death of father	20.Death of close family member	76.4	
Death of mother	20.Death of close family member	76.4	
Divorce	35.Divorce	64.7	
Occupational disability	03.Major injury/Illness to self	66.9	Closest category description
Unemployment	18.Fired/Laid-off/Unemployed	54.8	
Bereavement	30.Death of spouse/mate	83.2	
**Events in-between waves**			
Death of father	20.Death of close family member	76.4	
Death of mother	20.Death of close family member	76.4	
Death of brother	20.Death of close family member	76.4	
Death of sister	20.Death of close family member	76.4	
Death of son	20.Death of close family member	76.4	
Death of daughter	20.Death of close family member	76.4	
Death of grandchild	34.Death of a close friend	51.5	Closest category description; chosen to contrast to first line of family (parent, sibling, child) versus second line of family (grandchild).
Illness of partner	27.Major injury/illness family	69.3	
Illness of significant other	34.Death of a close friend	51.5	Closest category description; chosen to contrast illness partner to illness of others.
Victim of crime	29.Victim of crime	72.0	
Conflict	44.Disagreement over child support, etc.	50.0	Closest category description
Financial problems	39.Financial problems/difficulties	60.7	
Divorce	35.Divorce	64.7	
Bereavement	30.Death of spouse/mate	83.2	

Descriptive statistics for severity scores of life events

Severity scores were divided by the lowest assigned severity, i.e. 50.0 (Conflict), so that this event has a weight of 1.0 and the rest has a higher weight.

Table S2. Descriptive statistics of life events severity scores			
**Variable**	**n**	**Mean (SD) or %**	**Observed range**
**Total severity before baseline (1992)**	2069	3.8 (1.35)	0 – 8.7
**Total severity up to 1995**	2004	5.3 (1.85)	0 – 12.8
**Total severity up to 1998**	1623	6.5 (2.38)	1 – 18.3
**Total severity up to 2002**	1227	7.9 (3.07)	1.5 – 19.7
**Total severity up to 2005**	845	9.1 (3.30)	1.5 – 21.8
**Recent events 1992-1995**	2004	1.5 (1.31)	0 – 7.1
**Recent events 1995-1998**	1669	1.3 (1.25)	0 – 7.2
**Recent events 1998-2002**	1308	1.5 (1.54)	0 – 7.9
**Recent events 2002-2005**	921	1.4 (1.18)	0 – 6.5
**Recent events 2005-2008**	746	1.4 (1.24)	0 – 6.3

Severity scores: Effects of life events on depressive symptoms (mutually adjusted)

Table S3. Effect of standardized life events severity scores on depressive symptoms (log-transformed and standardized), adjusted for age, sex and baseline depressive symptoms				
**Variable**	**obs**	**B**	**95% C.I.**	***p***
**Severity of Proximate events**	6345	0.09	0.07 to 0.11	<.001
**Severity of Cumulative events**	6345	0.15	0.13 to 0.17	<.001

Severity scores: Two-way interaction effects with life events

Table S4. Two-way interaction effects between standardized cumulative life events severity and proximate life events severity, adjusted for age, sex and baseline depressive symptoms			
**Variable**	**B**	**95% C.I.**	***p***
**Cumulative and Proximate Events**			
Severity of proximate events	0.09	0.07 to 0.11	<.001
Severity of cumulative events	0.16	0.13 to 0.18	<.001
Severity of proximate * cumulative events	-0.02	-0.04 to -0.004	.02

Severity scores: Statistically significant three-way interaction effects with life events

Table S5. Three-way interaction effects between psychosocial factors and the interaction between standardized cumulative and proximate life events severity[a)]

Variable	B	95% C.I.	*p*
**Mastery, Proximate and Cumulative Events**			
Severity of proximate events	0.07	0.06 to 0.09	<.001
Severity of cumulative events	0.12	0.09 to 0.14	<.001
Mastery (standardized)	-0.30	-0.32 to -0.28	<.001
Severity of Proximate * Cumulative events	-0.01	-0.03 to 0.01	.17
Mastery*Severity of proximate events	-0.001	-0.02 to 0.02	.88
Mastery*Severity of cumulative events	0.01	-0.01 to 0.03	.41
Mastery * Severity of Proximate * Cumulative events	0.02	0.002 to 0.04	.03
**Neuroticism, Proximate and Cumulative Events**			
Severity of proximate events	0.09	0.07 to 0.11	<.001
Severity of cumulative events	0.15	0.13 to 0.17	<.001
Neuroticism (standardized)	0.30	0.27 to 0.33	<.001
Severity of Proximate * Cumulative events	-0.02	-0.03 to 0.002	.08
Neuroticism*Severity of proximate events	-0.01	-0.02 to 0.01	.56
Neuroticism*Severity of cumulative events	-0.03	-0.05 to -0.01	.01
Neuroticism * Severity of Proximate * Cumulative events	-0.02	-0.03 to 0.001	.06

a) adjusted for age, sex and baseline depressive symptoms

Conclusion of sensitivity analyses with severity scores:

Main results with severity scores are similar to results with event counts

Full results from models with statistically significant interaction effects between proximate and cumulative events and psychosocial factor

Mastery:

Table S6. Three-way interaction effects (p<.10) between mastery, proximate and cumulative life events on depressive symptoms (log-transformed and standardized) [a)]

		Composite effect			Within-person effect			Between-person effect		
**Variable**		**b**	**95% C.I.**	***p***	**b**	**95% C.I.**	***p***	**b**	**95% C.I.**	***p***
Constant		-0.34	-0.42 to -0.25	<.001	-0.51	-0.61 to -0.41	<.001	-0.10	-0.27 to 0.06	.21
Proximate events (ref=0)										
	1	0.07	-0.02 to 0.17	.14	0.17	0.06 to 0.28	.002	-0.14	-0.37 to 0.10	.26
	2	0.25	0.13 to 0.36	<.001	0.38	0.25 to 0.51	<.001	0.10	-0.16 to 0.37	.44
	3+	0.27	0.12 to 0.42	<.001	0.49	0.31 to 0.67	<.001	-0.13	-0.48 to 0.21	.45
Cumulative events		0.06	0.04 to 0.08	<.001	0.09	0.07 to 0.11	<.001	0.01	-0.04 to 0.05	.80
Mastery		-0.30	-0.38 to -0.22	<.001	-0.19	-0.29 to -0.10	<.001	-0.33	-0.50 to -0.16	<.001
Proximate * Cumulative										
	1	-0.002	-0.02 to 0.02	.88	-0.02	-0.04 to 0.01	.13	0.05	-0.02 to 0.12	.14
	2	-0.02	-0.05 to 0.003	.09	-0.04	-0.07 to -0.01	.003	0.001	-0.07 to 0.07	.98
	3+	-0.01	-0.04 to 0.03	.69	-0.04	-0.07 to -0.003	.03	0.08	-0.01 to 0.17	.10
Mastery * Proximate events										
	1	0.04	-0.06 to 0.14	.45	0.01	-0.10 to 0.12	.82	-0.001	-0.24 to 0.24	.99
	2	-0.08	-0.19 to 0.04	.21	-0.12	-0.25 to 0.01	.07	-0.01	-0.27 to 0.24	.92
	3+	-0.13	-0.28 to 0.02	.08	-0.21	-0.38 to -0.04	.01	-0.06	-0.38 to 0.26	.71
Mastery * Cumulative evts		-0.001	-0.02 to 0.02	.88	-0.01	-0.03 to 0.01	.51	-0.03	-0.08 to 0.02	.21
Mastery * Proximate events * Cumulative events										
	1	-0.004	-0.03 to 0.02	.70	-0.003	-0.03 to 0.02	.81	0.03	-0.04 to 0.10	.40
	2	0.02	-0.004 to 0.05	.10	0.03	-0.002 to 0.05	.07	0.03	-0.04 to 0.10	.38
	3+	0.03	<0.001 to 0.06	.05	0.04	0.01 to 0.07	.02	0.05	-0.03 to 0.13	.24

a) All models are adjusted for age, sex and baseline depressive symptoms. Mastery is standardized.

Neuroticism:

Table S7. Three-way interaction effects (p<.10) between neuroticism, proximate and cumulative life events on depressive symptoms (log-transformed and standardized)[a]				
		Composite effect		
**Variable**		**b**	**95% C.I.**	***p***
Constant		-0.43	-0.52 to -0.35	<.001
Proximate events (ref=0)				
	1	0.11	0.01 to 0.21	.03
	2	0.30	0.18 to 0.41	<.001
	3+	0.33	0.18 to 0.49	<.001
Cumulative events		0.08	0.06 to 0.10	<.001
Neuroticism		0.31	0.23 to 0.40	<.001
Proximate * Cumulative events				
	1	-0.01	-0.03 to 0.02	.53
	2	-0.03	-0.05 to -0.01	.02
	3+	-0.01	-0.04 to 0.02	.58
Neuroticism * Proximate events				
	1	0.07	-0.03 to 0.17	.15
	2	0.002	-0.12 to 0.12	.98
	3+	0.16	0.01 to 0.31	.04
Neuroticism * Cumulative events		-0.002	-0.02 to 0.02	.85
Neuroticism * Proximate events * Cumulative events				
	1	-0.02	-0.04 to 0.01	.15
	2	-0.01	-0.03 to 0.02	.56
	3+	-0.04	-0.07 to -0.01	.01

a) All models are adjusted for age, sex and baseline depressive symptoms. Neuroticism is standardized.

Table S8. comparison of results in samples excluding different types of drop-out						
**Effect**	**Total sample n=2069**		**Excluding deceased n=1045**		**Excluding all drop-out n=573**	
	Point estimate (b)	p-value	Point estimate (b)	p-value	Point estimate (b)	p-value
**Model without interactions (as in Table 2)**						
Proximate events						
1	0.08	<.001	0.11	<.001	0.14	<.001
2	0.18	<.001	0.21	<.001	0.23	<.001
3+	0.32	<.001	0.35	<.001	0.39	<.001
Cumulative events	0.07	<.001	0.07	<.001	0.06	<.001
**Model with 2-way interaction (as in Table 3)**						
Proximate*Cumulative events						
1	-0.01	.40	-0.01	.65	-0.01	.35
2	-0.03	.01	-0.03	.03	-0.04	.04
3+	-0.02	.17	-0.02	.24	-0.04	.11
**Models with significant (p<.10) composite three-way interactions (as in Table 4)**						
Proximate*Cumulative*Mastery						
1	-0.004	.70	-0.005	.71	0.002	.89
2	0.02	.10	0.02	.24	0.02	.20
3+	0.03	.05	0.03	.08	0.02	.40
Proximate*Cumulative*Neurot.						
1	-0.02	.15	-0.01	.36	-0.04	.05
2	-0.01	.56	0.004	.82	-0.02	.31
3+	-0.04	.01	-0.02	.17	-0.03	.19

Conclusion from sensitivity analyses with smaller sample selections:

The point estimates are generally comparable in the sample excluding deceased participants. Main effects of proximate events are somewhat stronger than in the full sample. Point estimates of the three-way interaction effect with neuroticism are weaker. p-values are generally higher, which is at least partly due to the decreased statistical power.

When further excluding all participants who did not have complete data across the measurement waves, point estimates are still generally comparable, yet p-values are substantially higher due to a large decrease in statistical power, which mainly affects the three-way interaction effects which need high power.

In sum, the effects of proximate events may have been somewhat underestimated in the total sample, yet the interaction effects seem to be robust to attrition.

Supplementary Figures

Figure S1. *Illustration of lagged effects of total event accumulation on depressive symptoms for the first three waves.*

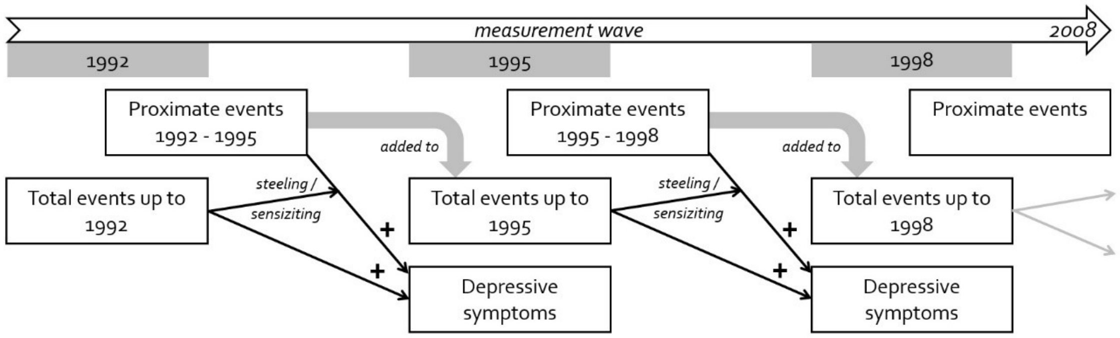

Figure S2. *Prevalence of life events, pooled across measurement waves.*
** indicates a statistically significant (p<.05) positive association with depressive symptoms*

A: Life events before baseline (1992/93; n=1780)

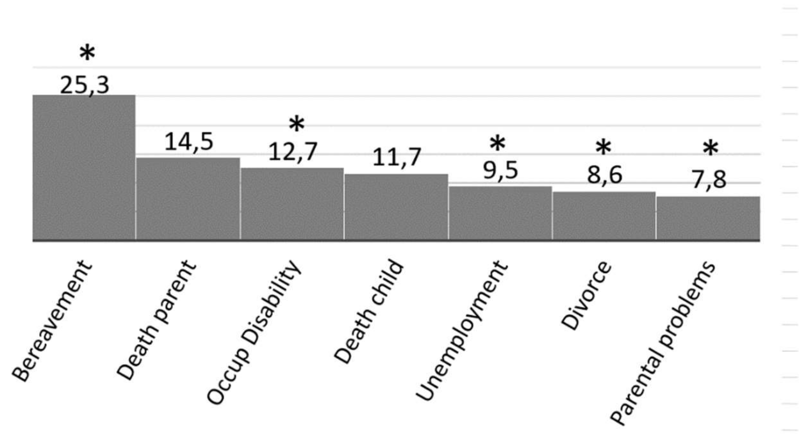

B: Life events in-between waves (proximate events) pooled (n=7494)

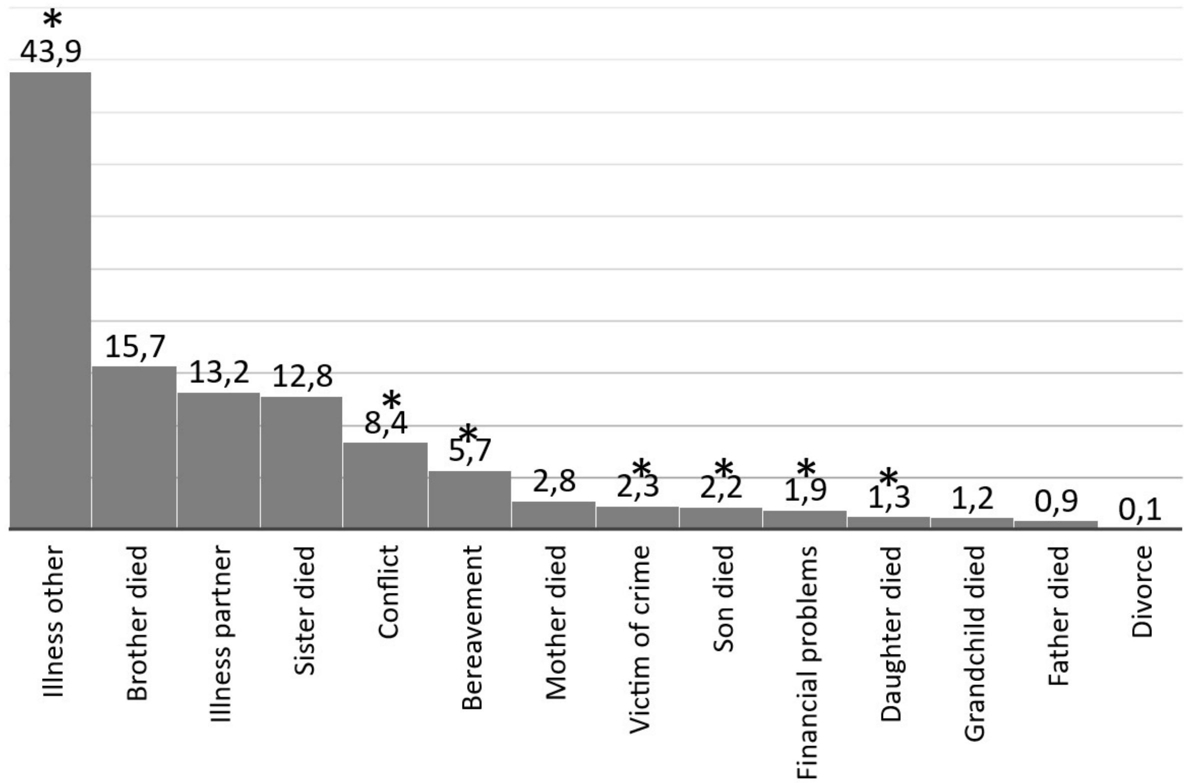

Figure S3. *Composite interaction effect between cumulative events and proximate events. Y-axis represents the level of depressive symptoms (log-transformed and standardized). Figure indicates that the difference in depressive symptoms between those with no proximate events and those with proximate events decreases as the number of cumulative events increases, suggesting a 'steeling' effect. Only the composite interaction effect between zero and 2 proximate events was statistically significant (p<.05).*

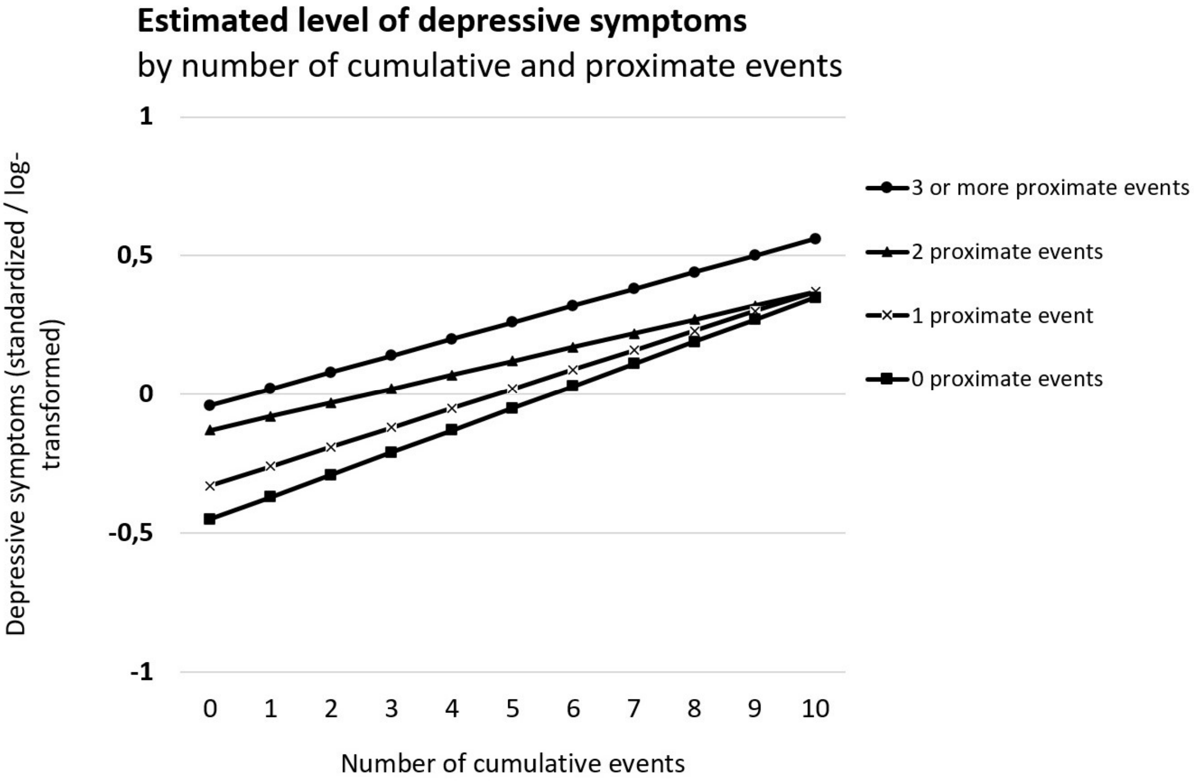

Supplement: gbab114_suppl_Supplementary_Materials [file gbab114_suppl_supplementary_materials.pdf]
